# Comparative Evaluation of *Prosopis cineraria* (L.) Druce and Its ZnO Nanoparticles on Scopolamine Induced Amnesia

**DOI:** 10.3389/fphar.2018.00549

**Published:** 2018-05-23

**Authors:** Ekta Yadav, Deepika Singh, Pankajkumar Yadav, Amita Verma

**Affiliations:** ^1^Bioorganic & Medicinal Chemistry Research Laboratory, Department of Pharmaceutical Sciences, Sam Higginbottom University of Agriculture, Technology & Sciences (SHUATS), Allahabad, India; ^2^Pharmaceutics Laboratory, Department of Pharmaceutical Sciences, Sam Higginbottom University of Agriculture, Technology & Sciences (SHUATS), Allahabad, India

**Keywords:** green synthesis, nanoparticles, acetylcholinesterase, Alzheimer’s, oxidative stress, behavioral models

## Abstract

Over recent years, utilization of green synthesized nanomaterials has been widely growing on human body because of its special properties. With the increasing acceptance of nanoparticle approach for various clinical treatments, the biosafety and toxicological effects on the vital organs such as central nervous system, have received more concern. Main focus of this study was to evaluate acute exposure of *n*-butanol fraction of *Prosopis cineraria* (L.) Druce hydroethanolic extract (BuPC) and green synthesized zinc oxide nanoparticles of BuPC (ZnOPC) on spatial cognition behavior, and to assess underlying mechanism by estimation of enzymatic antioxidative status along with acetylcholinesterase (AChE) activity in mice brain. Strongest *in vitro* antioxidant and AChE inhibitory activity exhibiting fraction, BuPC, was examined for inhibition kinetic study by Lineweaver–Burk and Dixon plots. BuPC was further used for fabrication ZnOPC and characterized by UV-visible spectroscopy, Fourier Transform Infrared (FTIR), Field Emission Scanning Electron Microscopy (FESEM), Energy Dispersive X ray (EDX), and Dynamic Light Scattering (DLS) analysis. Old male swiss albino mice were randomly divided into seven groups and treated for 21 days. Subsequently spatial memory was determined by two behavioral models [Elevated plus maze (EPM) and Hebbs William maze (HWM)] and supernatant of brain homogenate was analyzed for enzymatic antioxidant level and AChE inhibitory activity. Zinc content of blood plasma and brain was estimated. Results showed prolonged transfer latency (TL) and time taken to reach reward chamber (TRC) by scopolamine was not ameliorated by the ZnOPC group, whereas BuPC group showed significant reduction in scopolamine induced increase in TL and TRC compared to control and scopolamine treated groups. ZnOPC alleviated enzymatic antioxidant activity and AChE as compared to donepezil and BuPC treated groups. Study concludes that ZnOPC attenuated spatial learning and memory by increase in oxidative stress and decrease in AChE activity at both dose levels. Our results suggest that BuPC exhibited a strong neuroprotective effect on cognitive deficit mice and it may be employed as a strong substance for the treatment of dementia whereas the green synthesized ZnOPC was not proficient to reverse the memory impairment induced by scopolamine.

## Introduction

Dementia is a mental syndrome associated with different types of neurodegenerative disease, such as Alzheimer’s and Parkinson’s disease, vascular dementia and frontotemporal dementia, etc. (Singh et al., 2016). Clinicopathologic studies revealed that the most common cause of dementia is Alzheimer’s disease which is considered as the 6th driving reason for death in the United States (Alzheimer’s Disease Facts and Figures, 2017). Alois Alzheimer coined the term Alzheimer’s disease, which is an age linked heterogeneous and crippling brain syndrome associated with impairment in learning and memory process, loss of capability to perform social or professional activity independently and change in personality (Sutalangka et al., 2013; Awasthi et al., 2016; Lohan et al., 2017). Pathophysiological hallmark of this disease is deficiency in cholinergic neurotransmission due to hydrolysis of acetylcholine by acetylcholinesterase (AChE) in central nervous system, oxidative and neuroinflammatory stress, extracellular deposition of β amyloid plaques and formation of neuronal fibrillary tangles (Mathew and Subramanian, 2014; Xu et al., 2014; Rizvi et al., 2016). Thus, currently the approach using anticholinesterase (inhibit AChE enzyme results in increase in acetylcholine content) and antioxidants to reduce the oxidative stress are the promising strategy for the treatment of dementia (Singh et al., 2013; Elnaggar et al., 2015). Since already existing approaches provide symptomatic relief and having inevitable side effects encouraged us to use herbals, which are already existing gift from the lap of nature with less side effects and more effectiveness.

*Prosopis cineraria* L. Druce (PC) also known as king of dessert belongs to Fabaceae family and a plant of Indian traditional medicine, used for management of various ailments. It is evergreen, small to moderate size tree, distributed in dry and arid region of India, mainly Rajasthan, Haryana, Gujarat and Punjab, as well as Oman, Afghanistan, and Pakistan (Karim and Azlan, 2012; Maideen et al., 2012; Jinu et al., 2017). It is locally known as khejri (Hindi), jandi (Urdu), shami or khijda (Sanskrit) (Ukani et al., 2000). Traditionally this plant has been used for the treatment of leucoderma, asthma, tremors, piles, bronchitis, eye disease, leprosy, boils, blisters, and mouth ulcers. Furthermore, different parts of plant possess laxative, antirheumatic, antidysenteric properties and reputed as a natural therapy for snake bite and scorpion sting (Robertson et al., 2011; Mohammad et al., 2013). It is also used for removal of hair from the skin by using wood ash (Sharma et al., 2010). Tribal people used flowers of the plant with sugar to prevent miscarriage during pregnancy, whereas stem bark is consumed for memory enhancement and to increase the concentration power of mind (Bithu et al., 2012). Different parts of PC are known to possess a number of therapeutic activities, e.g., antihyperglycemic, antihypercholesteremic (Sharma et al., 2010), antitumour (Robertson et al., 2011), analgesic (Kumar et al., 2011), antibacterial (Neghabi-Hajiagha et al., 2016), antioxidant and used in gastrointestinal, respiratory, and vascular disorders (Janbaz et al., 2012). Literature study revealed that PC contains a wide range of secondary metabolites such as flavonoids, phenolic compounds, alkaloids along with some isolated compounds as diketones, patulitrin, spicigerin, β-sitosterol, stigmasterol, octasanol, hentriacontane, and prosogerin A, B, C, and D (Ukani et al., 2000; Malik and Kalidhar, 2007).

In recent years, green synthesis of nano particles have been grabbing more attention of researchers over traditional chemical method, since it does not need any additional capping agent which leads to formation and absorption of several toxic chemicals and ultimately alters the therapeutic application (Kharissova et al., 2013). In green synthesis method, metal oxide nanoparticles are fabricated by using several non-toxic and ecofriendly biological methods with the help of whole plant extract, enzymes, bacteria or algae, as it consists bioactive secondary metabolites (Savoia, 2012; Mittal et al., 2013; Janaki et al., 2015). It is easy to prepare, economic, reproductive, readily scalable and high yielding method (Ramesh et al., 2015; Yuvakkumar et al., 2015). Green synthesized nanoparticles of PC leaf has been investigated on inhibition of MCF-7 cancer cells (Jinu et al., 2017), while cytotoxicity of nanoparticles of PC leaf and bark on two human cancer cell lines, i.e., HeLa and MCF-7, was evaluated by Roberson et al. (2014).

Zinc (Zn) is the second most abundant essential micronutrient in the human body which is necessary for DNA replication, transcription, protein synthesis, cell growth and division. Dietary Zn deficiency may cause learning impairment as a result of alteration in Zn homeostasis in the brain (Takeda, 2000). Zn play a vital role in the safeguarding of blood brain barrier (BBB) against free radical induced oxidative stress and necessary for coenzymes synthesis which assist in biogenic amines production and metabolism (Amara et al., 2014). Literature study revealed that along with the use of chemically synthesized Zn oxide nanoparticles (ZnONP), use of green synthesized ZnONP also growing rapidly, which results in increase of human exposure to nanoparticles suggesting strict need to fundamentally understand its effect on human vital organs, i.e., central nervous system.

Literature also advocates lack of studies that could clear the pathophysiological effect of green fabricated ZnONP on *in vivo* oxidative stress, which ultimately results in cognitive impairment and memory loss via cell injury and neuronal death, as well as on other brain functions (Maruyama et al., 2006; Ademosun et al., 2015). Thus, there is a need to know about advantages, disadvantages, ideal approaches and their limitations regarding the use of green synthesized ZnONP in nanomedicine. Extensive use of metal oxide nanoparticles and their possible threat to central nervous system has been alarming situation recently. Concerning this, key objective of the present study was to evaluate the potential of folklore medicinal plant extract fraction and green synthesized ZnONP of PC (ZnOPC) on cognitive behavior via analysis of oxidative defense system and estimation of key enzyme levels in scopolamine induced cognitive deficit mice models.

## Materials and Methods

### Chemicals and Reagents

Scopolamine hydrobromide, 1, 1-diphenyl-2-picrylhydrazyl (DPPH), 5,5′-dithio-bis-(2-nitro) benzoic acid (DTNB), acetylthiocholine iodide (ATCI), AChE, rutin and gallic acid were purchased from Sigma-Aldrich (St. Louis, MO, United States). Berberine chloride, ascorbic acid, ethylene di amine tetra acetic acid (EDTA) and nitro blue tetrazolium (NBT) were procured from HiMedia Laboratories Pvt. Ltd., Mumbai. Folin-Ciocalteu’s phenol reagent was obtained from Merck KGaA (Darmstadt, Germany). All solvents and reagents used in our study were of analytical grade.

### Collection of Plant Material

Fresh leaves of PC were procured from the rural area of Rewari, Haryana (India) in June 2015. The plant was identified and authenticated by botanist Dr. R. M. Kadam, Department of Botany, Mahatma Gandhi Mahavidyalaya, Latur, Maharashtra, India. The voucher specimen (DI 15) of plant has been deposited in the herbarium of same institution.

### Hydroethanolic Extract Preparation and Fractionation

Fresh leaves of plant (5 kg) were collected, washed with water, drained, shade dried and coarsely powdered. Dried powder was extracted with petroleum ether (60–80°C) to remove fatty material. Defatted plant material was macerated to prepare hydroethanolic extract (ethanol: water, 70:30). Maceration procedure repeated thrice, filtered and all three filtrates were combined. Filtrate was dried under vacuum to obtain the hydroethanolic extract (551 g).

Dried extract was subjected to fractionation by liquid-liquid partition in increasing order of polarity. The hydroalcoholic extract was dissolved in water, transferred into a separating funnel and then partitioned with chloroform, ethyl acetate and *n*-butanol, respectively. All three resultant fractions, i.e., chloroform (CHFCP, 98.6 g), ethyl acetate (EAPC, 75.2 g) and *n*-butanol (BuPC, 132.2 g), separately collected, dried under reduced pressure and preserved at -20°C.

### Colorimetric Determination of Total Phenolic Compounds

Total phenolic compounds content of crude hydroethanolic extract of PC was estimated by Folin-Ciocalteu method (Mohamed et al., 2016). The result was determined from calibration curve of standard solution of gallic acid and expressed as mg gallic acid equivalent (GAE)/g of extract. The experiment was repeated thrice.

### Colorimetric Determination of Total Flavonoid Compounds

Total flavonoid content was determined by aluminum chloride colorimetric technique and expressed as mg rutin equivalent (RE)/g of extract from standard rutin solution calibration curve (Laghari et al., 2011). The experiment was repeated thrice.

### DPPH Radical Scavenging Activity

Ability of PC fractions (CHFPC, EAPC, and BuPC) to donate hydrogen or electron was estimated by colorimetric method using stable free radical DPPH (Kedare and Singh, 2011). Briefly, reaction mixture was prepared by adding 150 μL of DPPH methanolic solution (3.3 mM) into 100 μL of PC fractions prepared in different concentrations. Each sample was scanned spectrophotometrically at 517 nm after 30 min and ascorbic acid was used as a standard. Potential of an antioxidant was measured by purple color bleaching reaction and decrease in absorbance, calculated by following formula:

$$\begin{array}{c} \mathrm{Activity}\;\mathrm{of}\;\mathrm{radical}\;\mathrm{scavenging}\;(\%)\;=\frac{\mathrm{Absorbance}\;\mathrm{of}\;control-Absorbance\;\mathrm{of}\;\mathrm{sample}}{\mathrm{Absorbance}\;\mathrm{of}\;\mathrm{control}}\times 100 \end{array}$$

### *In Vitro* AChE Inhibitory Activity of PC Fractions

All three fractions of PC were subjected to determination of their ability to inhibit AChE by using Ellman’s assay with slight modifications (Sugimoto et al., 1995; Tung et al., 2017). Reaction was started with introduction of 140 μL of sodium phosphate buffer (0.1 M), 20 μL of sample (prepared in DMSO) and 20 μL of AChE (0.25 IU/ml). After placing the resultant mixture at room temperature for 15 min, added 10 μL of DTNB (2.5 mM) and 10 μL ATCI (2.0 mM) and incubated it for 10 min at 25°C. Absorbance of each mixture was measured at 412 nm. To compare the inhibition of AChE activity, berberine chloride was used as a positive control and percentage inhibition of AChE was calculated by following formula:

$$\begin{array}{c} \mathrm{Inhibition}\;\mathrm{of}\;\mathrm{AChE}\;(\%)=\frac{\mathrm{Absorbance}\;\mathrm{of}\;control-Absorbance\;\mathrm{of}\;\mathrm{sample}}{\mathrm{Absorbance}\;\mathrm{of}\;\mathrm{control}}\times 100 \end{array}$$

### Kinetics of AChE Inhibition

BuPC fraction was used to analyze the kinetics of inhibition by using previously described method (Kamal et al., 2000). Assay was started with introduction of 140 μL sodium phosphate buffer (0.1 M, pH 8.0) in various concentrations (0, 20, 40, and 80 μg/ml) of 20 μL of BuPC followed by addition of 20 μL of AChE (0.25 IU/ml), stirred and incubated for 15 min at room temperature. 10 μL of DTNB (2.5 mM) and 10 μL of different concentrations (1.0, 2.0, and 4.0 mM) of ATCI were mixed in it and color intensity was measured at 412 nm during 5 min. Each procedure was repeated thrice. To analyze the type of AChE inhibition kinetics, Lineweaver–Burk graph was plotted between 1/[ATCI] and 1/(Absorbance/min). Inhibition constant (*K*_i_) was calculated from intercept in Dixon plot {concentration of BuPC vs. 1/(Absorbance/min)}.

### Fabrication of ZnONP

Preparation of nanoparticles using plant extract is known as green synthesis. Co-precipitation method was used (Vijayakumar et al., 2016). 1 g of BuPC was dissolved in 1 ml of DMSO. 200 μL of resultant solution was mixed slowly with constant stirring into 50 ml of Zn acetate dihydrate (2 M) prepared in distilled water. 50 ml of NaOH (2 M) was introduced into it and placed on a magnetic stirrer for about 2 h. Precipitate obtained from the reaction mixture by centrifugation at 12000 rpm for 15 min was repeatedly washed with water followed by ethanol to remove impurities and dried overnight at 60°C in oven. Calcination of resultant powder was performed with the help of muffle furnace for 3 h at 360°C.

### Characterization of ZnOPC

#### UV Spectrophotometry

Optical characteristics of BuPC and ZnOPC were observed with the help of UV-Visible spectrophotometer (UV-2450, Shimadzu). The synthesized sample was made ready for scanning by re-suspending in sterile de-ionized water.

#### Fourier Transform Infrared (FT-IR)

Spectrum was recorded using FT-IR spectrophotometer to identity the presence of various bioactive phytoconstituents which are known to be responsible for reduction and stabilization of ZnONP (Perkin-Elmer Spectrum 1000). FT-IR spectra were obtained at room temperature by using KBr pallets of BuPC and ZnOPC in the wavelength range of 4500–400 cm^-1^.

#### Field Emission Scanning Electron Microscopy (FESEM)

The FESEM (Carl Zeiss, Germany) associated with energy dispersive X-ray spectroscopy (EDX-JEOL, JSM-5610) was used to identify the surface morphology and metal analysis of synthesized ZnOPC. Dry sample powder was sprinkled over the carbon tape and then coated with gold for FESEM analysis.

#### Dynamic Light Scattering (DLS)

Sample was suspended in sterile de-ionized water and then analysis was performed by using Zetasizer Nano (DLS, Malvern Instruments, Worcestershire, United Kingdom) just after sonication for 15 min. Zetasizer was equipped with a He/Ne red laser at 633 nm wavelength along with a detector set at 173° which results in size measurement with high sensitivity. Hydrodynamic diameter, zeta potential and polydispersity index (PdI) were determined as a function of time.

### Animals

Male swiss albino mice (8 weeks old) of 25–30 g weight were used for evaluation of antiamnesic activity. Throughout the experimental duration, mice were kept in polypropylene cages in well maintained room with standard laboratory conditions, i.e., temperature (25 ± 2°C), relative humidity (55–60%), 12:12 light and dark cycle. Animals were freely accessible to water and balanced rodent pellet food *ad libitum*. Before starting the behavioral experiment, mice were acclimatized to laboratory conditions for 7 days. The Institutional Animal Ethics Committee (IAEC) approved the experimental protocol (Approval Number IAEC/SHIATS/PA16III/SEYAV09).

#### Animal Experimental Protocol

All mice were randomly divided into seven groups comprising nine animals in each group.

Group I (Control): Normal saline at dose level of 10 ml/kg/day p.o.
Group II (SCO): Vehicle and scopolamine at dose level of 0.6 mg/kg/day i.p.
Group III (DON): Donepezil at dose level of 1 mg/kg/day p.o.
Group IV (BuPC400): BuPC at dose level of 400 mg/kg/day p.o.
Group V (BuPC600): BuPC at dose level of 600 mg/kg/day p.o.
Group VI (ZnOPC15): Green synthesized ZnOPC at dose level of 15 mg/kg/day p.o.
Group VII (ZnOPC30): Green synthesized ZnOPC at a dose level of 30 mg/kg/day p.o.

All animals were treated with above mentioned drug regime, whereas vehicle was administered to Group II for 21 days. On last day (day 21), all groups (2–7), except group I, were administered with scopolamine (0.6 mg/kg i.p.) after 60 min of respective treatment for induction of memory deficit. After 30 min of scopolamine injection, acquisition trials were performed on all groups and retention of memory was examined after 24 h, on day 22. After being tested by one behavioral model, animals were provided a washout period of 15 days for testing by another model.

### Behavioral Tests

#### Elevated Plus Maze (EPM)

Elevated plus maze is an exteroceptive behavioral model widely used for evaluation of potency of various drugs on learning and memory in rodents. It consists of two open arms (16 cm × 5 cm) and two covered arms (16 cm × 5 cm × 12 cm) extended from a central platform (5 cm × 5 cm). Height of the maze was 25 cm from the floor (Itoh et al., 1990). On training day (day 21), after 30 min of administration of scopolamine injection, each mouse was kept at the end of an open arm, facing away from the central platform. Transfer latency (TL) is known as the index for learning and memory process, defined as the time taken by mice to move from the open arm to one of closed arm with its four legs was noted for each mouse. 90 s TL was allotted but if animal did not search for the covered arm within 90 s of its placement, it was quietly directed into one of the two covered arm. Another 2 min was given to each mouse to explore the maze and then transferred back to its cage. After 24 h of training period, retention of memory was studied by recording the TL. Significantly reduced TL shows memory enhancing effect. To ensure consistency of experiment prior to the test session, animals were brought in the testing room 1 h prior to the start of behavior testing. Test room lighting, temperature and noise level were kept constant for all mice (Mani et al., 2011).

#### Hebbs William Maze (HWM)

Hebbs William maze (HWM) is an incentive-based exteroceptive behavioral model useful for measurement spatial working memory of rodents. Structure of HWM consists of three components, (i) animal chamber, extended into (ii) the middle chamber (exploratory area) and (iii) a reward chamber at the other end of maze, where food was placed as a reward. Guillotine removable doors were used in all three compartments. On 1st day (day 21), after drug treatment protocol, mouse was placed individually in animal chamber, keeping the door opened to facilitate the entry into the next chamber. As animal moved into next chamber, door of the start box was immediately closed to prevent back entry. Time taken by the animal to reach reward chamber (TRC) from start box on 1st day, reflects the learning index, was recorded. Before shifting to home cage, each animal was allowed to explore the maze for another 3 min with all the doors open. Retrieval of memory of this training task was examined after 24 h of the 1st-day trial. Significant reduction in TRC value indicates improvement of memory (Parle and Bansal, 2011).

### Collection of Blood, Brain Tissue and Tissue Homogenate Preparation

After behavioral test, all mice from every group were sacrificed by cervical dislocation. Trunk blood was collected and preserved into tubes containing EDTA as an anticoagulant. With great care, whole brain were quickly removed and weighed. Brain tissues of each group were processed for biochemical estimation, Zn content determination and for histological examination. Isolated brain was homogenized for biochemical estimation in ice cold sodium phosphate buffer (pH 7.4). The brain homogenate was then centrifuged at a speed of 14500 rpm for 15 min at 4°C. Resultant clear supernatant was taken for lipid peroxidation index, antioxidant enzyme assay and AChE activity in brain.

### Estimation of Brain AChE Activity

AChE activity in the mice brain was measured by using slightly modified Ellman’s method, as described above, using ATCI as a substrate. Reaction was initiated by pouring 0.1 M sodium phosphate buffer and 2.5 mM DTNB followed by addition 2 mM ATCI in a volumetric flask. Then, brain homogenate supernatant was added into the prepared reaction mixture and incubated at 30°C for 10 min. This resulted into the formation of yellow color owing to DTNB reduction by certain substrates in brain homogenate and non-enzymatic hydrolysis of other substrates. The sample was observed spectrophotometrically at 412 nm for change in absorbance and activity was represented as nM/min/mg protein (Hritcu et al., 2015).

### Estimation of Lipid Peroxidation Index

Thiobarbituric acid reactive substance (TBARS) is considered as a marker of lipid peroxidation. The extent of lipid peroxidation was estimated by determining the concentration of thiobarbituric acid in brain (Yagi, 1976). Assay mixture was prepared by addition of 700 μL of phosphoric acid (9%) and 250 μL of thiobarbituric acid in 250 μL of supernatant and shaken gently. The mixture was boiled for 60 min and after cooling 1250 μL butanol added. Whole mixture was agitated for 20 s by vortex mixer and finally centrifuged for 20 min at 25°C. Absorbance of the solution was measured at 534 nm. Results are expressed as nM/mg protein.

### Determination of Antioxidant Enzyme Activity

#### Superoxide Dismutase (SOD) Activity

Brain SOD activity was measured according to method described by Winterbourn et al. (1975), based on principle of photoreduction of NBT dye by SOD enzyme. Reaction mixture was made ready with 1.5 ml solution of 100 mM Tris-HCl buffer of pH 7.8, 75 mM NBT, 2 μM riboflavin and 6 mM EDTA. 700 μL of reaction mixture was introduced into 100 μL of supernatant. Color intensity of the mixture was measured at 560 nm and enzymatic activity expressed as units/mg protein.

#### Catalase (CAT) Activity

Assay was started with the addition of 100 μL supernatant into 150 μL phosphate buffer (0.01 M) having pH 7.0 followed by addition of 250 μL H_2_O_2_ (0.16 M). Resultant reaction mixture was then incubated for 1 min at 37°C and the assay was completed by pouring of 1 ml of reagent (dichromate: acetic acid). Green color was formed after boiling the reaction mixture for 15 min and then absorbance was examined spectrophotometrically at 570 nm (Sinha, 1972). CAT activity is represented as μM of H_2_O_2_ consumed/min/mg protein.

#### Glutathione Peroxidase (GPx) Activity

Assay was performed by DTNB method described by Sharma and Gupta (2002). 200 μL of supernatant was introduced into prepared reaction mixture composed of 1 ml phosphate buffer (0.4 M, pH 7.0), EDTA (0.4 mM), 1 ml of NaN_3_ (5 mM) and 1 ml of glutathione (4 mM). 1 ml of H_2_O_2_ (4 mM) was added after incubating the mixture at 37°C for 5 min. Glutathione concentration was determined by recording the absorbance of solution with the help of spectrophotometer at 412 nm.

### Determination of Zn Content

Amount of Zn was evaluated in brain and blood plasma of treated animals. Plasma was collected after centrifugation of collected blood samples. Carefully isolated brains were immediately weighed, stored in dry ice and then digested in concentrated HNO_3_ for 3 h at 160°C. Atomic absorption spectrometer (Avanta-R, GBC, Australia) was used to analyze Zn concentration in brain and plasma with the help of acetylene gas as fuel and air as an oxidizer. Concentration of Zn was determined from the calibration curves (prepared separately with suitable concentrations of the standard solution of Zn). Detected Zn concentration was up to the limit of 0.05 ppm and expressed as ng/g of the dry mass of brain and μg/ml of plasma.

### Histological Studies

Isolated brain tissues from all groups were immediately stored in 10% formalin solution. After 24 h, tissue was subjected to dehydration with gradual concentration of ethanol. Dehydrated brain tissue was cleaned by xylene and entrenched in paraffin wax. Brain sections of 0.5 μm thickness were prepared and then stained with hematoxylin and eosin dye for photomicroscopic observation (Drury, 1983).

### Statistical Analysis

All results were represented as mean ± standard deviation. Data were statistically analyzed by one-way ANOVA followed by Newman–Keuls multiple comparison test. Value of *p* < 0.05 was considered as statistically significant.

## Results

### Evaluation of Total Phenolic and Flavonoid Contents

Total phenolic contents of PC were found to be 123.12 ± 1.43 mg of GAE/g of dry extract. The amount of flavonoid content was estimated to be 145.17 ± 0.32 mg of RE/g of dry extract.

### *In Vitro* Antioxidant Activity of PC Fractions

**FIGURE 1:**
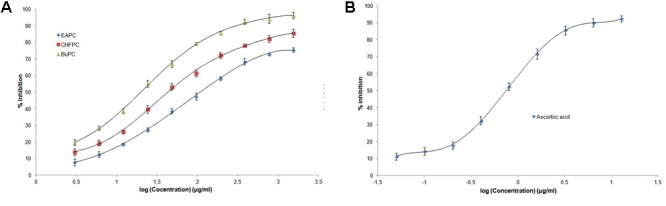
*In vitro* antioxidant activity by DPPH method for **(A)** different fractions of PC hydroethanolic extract and **(B)** ascorbic acid. Data represented as mean (*n* = 3) ± SD.

Free radical scavenging ability of all fractions was evident of their antioxidant activity assessed by DPPH inhibition at 517 nm as shown in **Figure 1A**. Data summarizes highest antioxidant activity by ascorbic acid (**Figure 1B**) followed by BuPC and CHFPC with IC_50_ value 0.74 ± 0.07, 19.85 ± 1.34, and 42.80 ± 2.23 μg/ml, respectively (**Table 1**). Least DPPH inhibition was obtained in EAPC (IC_50_ value of 107.82 ± 1.71 μg/ml). Antioxidant activity of all fractions was observed to be dose dependant.

**Table 1:** Antioxidant activity of different fractions of PC extract and ascorbic acid.

Sample	Log IC_50_ (μg/ml)	IC_50_ (μg/ml)
EAPC	2.0327	107.82 ± 1.71
CHFPC	1.6315	42.80 ± 2.23
BuPC	1.2979	19.85 ± 1.34
Ascorbic acid	–0.1258	0.74 ± 0.07

### *In Vitro* AChE Inhibition by PC Fractions

**FIGURE 2:**
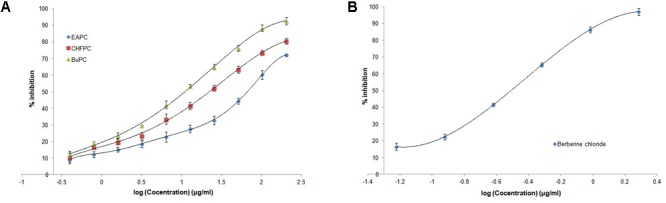
*In vitro* AChE inhibitory activity by Ellman’s method for **(A)** different fractions of PC hydroethanolic extract and **(B)** berberine chloride. Data represented as mean (*n* = 3) ± SD.

IC_50_ values of different fractions of hydroethanolic extract of PC and berberine chloride on AChE inhibition is shown in **Table 2**. **Figure 2A** indicates the concentration dependant effect of all fractions of hydroethanolic extract of PC on inhibition of AChE. Berberine chloride (**Figure 2B**) and BuPC showed the highest inhibitory activities than other fractions with IC_50_ value of 0.30 ± 0.03 μg/ml and 11.06 ± 3.61 μg/ml, respectively, followed by CHFPC (22.35 ± 2.73 μg/ml). Lowest AChE inhibitory activity (IC_50_ value of 66.88 ± 3.45 μg/ml) was observed in EAPC.

**Table 2:** Inhibition of AChE by different fractions of PC extract and berberine chloride.

Sample	Log IC_50_ (μg/ml)	IC_50_ (μg/ml)
EAPC	1.823	66.88 ± 3.45
CHFPC	1.349	22.35 ± 2.73
BuPC	1.043	11.06 ± 3.61
Berberine chloride	–0.508	0.30 ± 0.03

### Kinetics of AChE Inhibition

**FIGURE 3:**
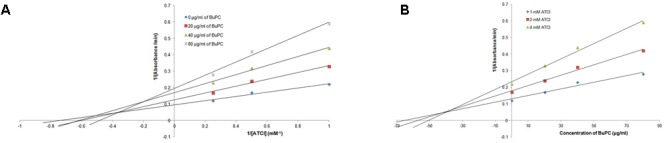
Graphical determination of kinetic (inhibition) type for BuPC by **(A)** Lineweaver–Burk plots and **(B)** Dixon plots. Data represented as mean (*n* = 3) ± SD.

The BuPC showed strongest *in vitro* AChE inhibition therefore, it was further evaluated for kinetic study. **Figure 3A** shows the kinetics of AChE inhibition by BuPC via Lineweaver–Burk plots [slope = *K*_m_/*V*_max_ and intercept with *X*-axis = (-1/*K*_m_)]. It represents the mixed kinetics and competitive inhibition. *K*_i_ constant signified the binding affinity between enzyme and inhibitor. It was taken into account from the point of intersection of three lines toward the negative values of the *X*-axis in Dixon plots. From **Figure 3B**, *K*_i_ value was found to be 39.82 ± 1.17 μg/ml.

### Characterization of ZnONP

#### UV Spectrophotometry

**FIGURE 4:**
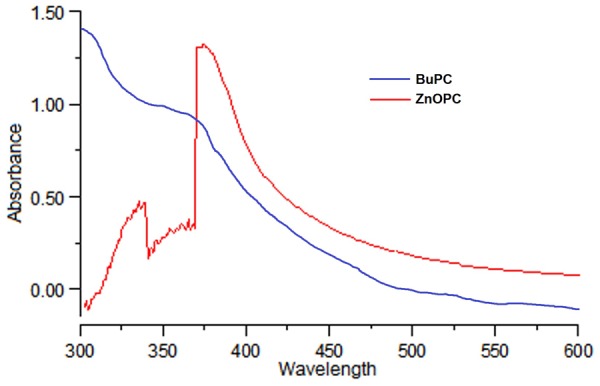
UV-Visible spectra of BuPC and green synthesized ZnOPC.

Since BuPC fraction showed the strongest *in vitro* antioxidant activity and AChE inhibition, it was further utilized to fabricate ZnOPC. UV–visible absorption spectra of BuPC and green synthesized ZnOPC given in **Figure 4**. ZnOPC showed a characteristic surface plasmon resonance absorption band at 374 nm, subjected to the absorption of ZnONP into the intrinsic band-gap due to transition of electron from the valence band to the conduction band. The absorbance at 374 nm signified the formation of ZnONP, which is well concordant with reported work (Rajiv et al., 2013).

#### Fourier Transform Infrared (FT-IR)

**FIGURE 5:**
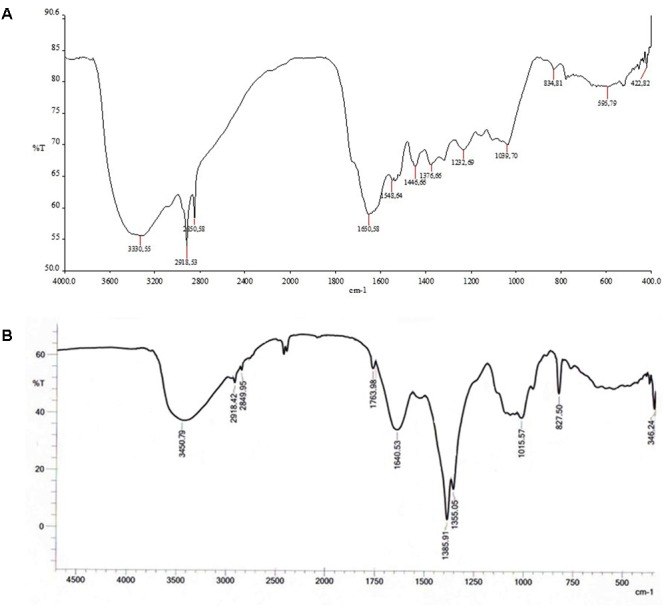
Fourier transform infrared (FT-IR) spectrogram of **(A)** BuPC and **(B)** green synthesized ZnOPC.

Fourier transform infrared (FT-IR) technique identifies the specific bond vibration peak owing to the reduction and capping of ZnONPs via probable secondary metabolites of plant extract. FT-IR spectra of BuPC exhibited characteristic peaks, i.e., 3330.55, 2918.53, 1650.58, 1446.66, 1376.66, and 1232.69 cm^-1^ which are attributed to existence of various functional groups such as -OH stretching of alcohols and phenols, -CH stretching of alkanes, C = O vibration, -CH_3_ bending vibration, C = C stretching of aromatic amines and -C = O groups of aromatic ring (**Figure 5A**). Spectrum of ZnOPC showed bands at 3450.79, 2918.42, 1763.98, 1640.53, 1385.91, 1355.05, 1015.57, 827.50, and 346.24 cm^-1^ (**Figure 5B**) which were assigned to –OH stretching of alcohols and phenols, -CH stretching of alkanes, C = O stretching, C = C stretching of aromatic amine, C–N stretching vibration of amine, C-H bending of alkane group (Bala et al., 2015; Nagajyothi et al., 2015; Ramesh et al., 2015). Hence, present FT-IR peaks are in agreement with some previous results of presence of rich amount of phytochemical compounds such as phenolic compounds, flavonoids, alkaloids, and terpenoids in ZnOPC (Malik and Kalidhar, 2007).

#### Field Emission Scanning Electron Microscopy (FESEM)

**FIGURE 6:**
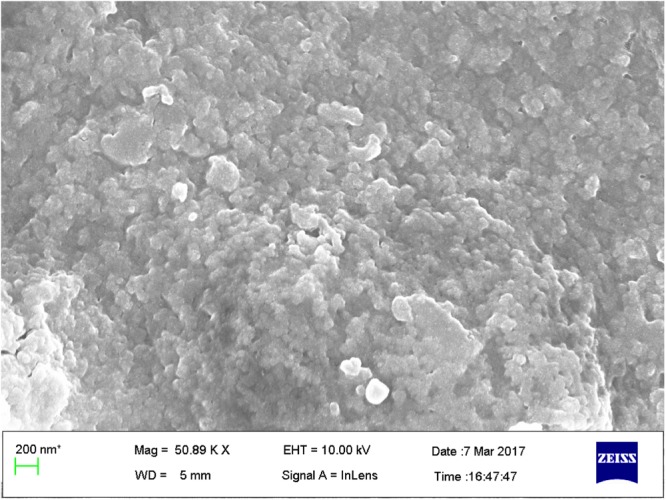
Field Emission Scanning Electron Microscopy (FESEM) micrograph of green synthesized ZnOPC.

ZnOPC surface morphology was observed with the help of FESEM micrographs. Photomicrographs (**Figure 6**) on higher magnification showed cauliflower like structure, made up of cluster of small and almost spherical structure having size range of 40–80 nm with rough surface.

#### Energy Dispersive X Ray (EDX)

**FIGURE 7:**
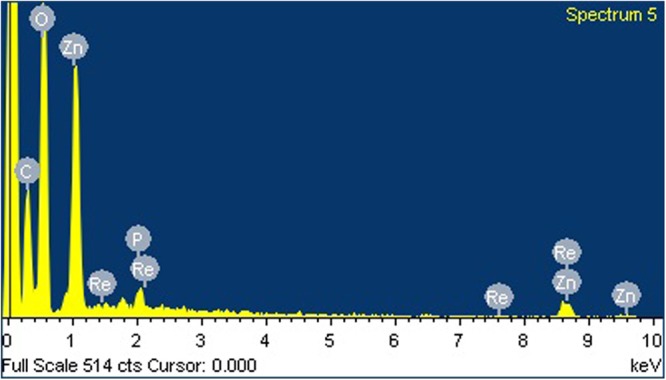
Energy dispersive X ray (EDX) spectra of green synthesized ZnOPC.

The metal composition in the nano structured sample was observed by EDX. **Figure 7** represents the presence of strong signals of Zn and oxygen atom in EDX spectra. Few other metal elements, such as carbon, phosphorous and rhenium were also observed in the EDX spectra. It indicates the surface adsorption of biologically active phytoconstituents from BuPC on the surface of ZnONP as stabilizing agents.

#### Dynamic Light Scattering (DLS)

**FIGURE 8:**
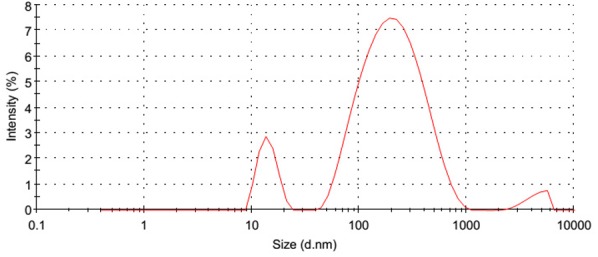
Dynamic light scattering (DLS) analysis of green synthesized ZnOPC.

Dynamic light scattering technique is basically utilized for determination of the hydrodynamic diameter of nanoparticles in suspension on the basis of their Brownian movements. The average hydrodynamic diameter was observed with DLS is 117.5 nm (**Figure 8**) which is relatively larger as compared to the sizes reported by FESEM. The nanoparticles size variation could be due to the characteristic of polydispersity which is considered in terms of polydispersity index value of 0.565. Polydispersity is an indicator of a distribution defines the degree of non-uniformity could be as a result of aggregation of nanoparticles which is attributed with the variation in calculated particle size than the actual size of the particles. Zeta potential was found to be -12 mV.

### Effect on TL in EPM

**FIGURE 9:**
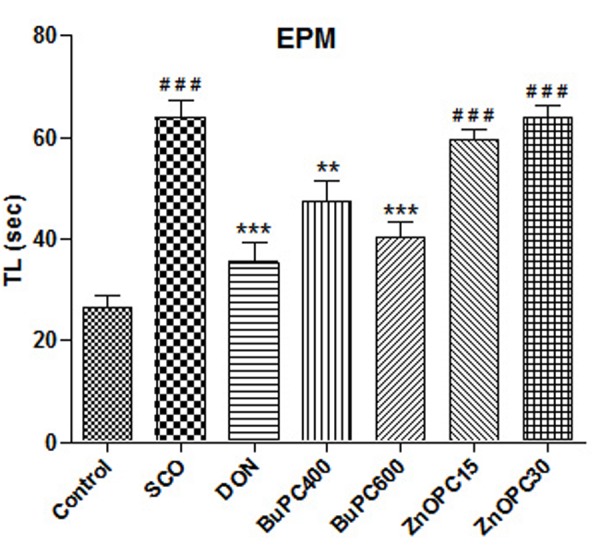
Effect of BuPC and ZnOPC on scopolamine induced memory impairment in mice using EPM model. Data represented as mean TL (s) ± SEM (*n* = 9), analyzed by one way ANOVA followed by Newman–Keuls multiple comparison test. Significant difference ^#^*p* < 0.05, ^##^*p* < 0.01, ^###^*p* < 0.001 in comparison to control group. Significant difference ^∗^*p* < 0.05, ^∗∗^*p* < 0.01, ^∗∗∗^*p* < 0.001 in comparison to scopolamine treated group.

In the EPM model, significant overall differences between groups [*F*(6,56) = 23.46, *p* < 0.0001] on the reference memory were shown in **Figure 9**. Scopolamine (*p* < 0.001) administered group showed significantly higher TL than control group during retention trial on day 22. Newman–Keuls multiple comparison test revealed that BuPC600 group (*p* < 0.001) significantly reduced TL, similar to DON group (*p* < 0.001), followed BuPC400 group (*p* < 0.01), shows reversal of amnesia induced by scopolamine. No significant change in TL (*p* > 0.05) by ZnOPC at both dose levels (15 and 30 mg/kg) indicates that it was not able to reverse the scopolamine induced amnesia.

### Effect on TRC in HWM

**FIGURE 10:**
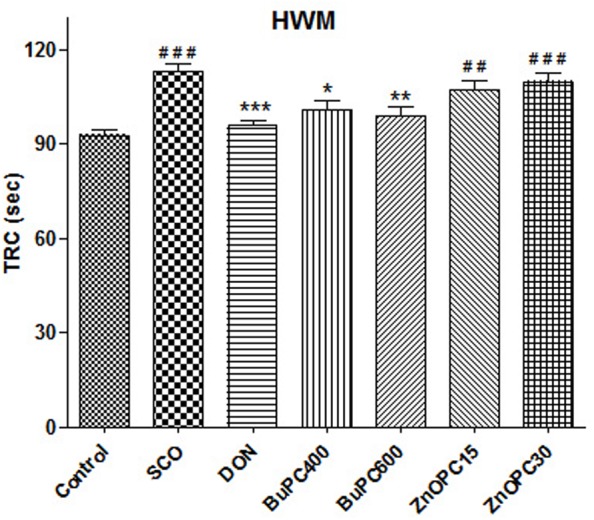
Effect of BuPC and ZnOPC on scopolamine induced memory impairment in mice using HWM model. Data represented as mean TRC (s) ± SEM (*n* = 9), analyzed by one way ANOVA followed by Newman–Keuls multiple comparison test. Significant difference ^#^*p* < 0.05, ^##^*p* < 0.01, ^###^*p* < 0.001 in comparison to control group. Significant difference ^∗^*p* < 0.05, ^∗∗^*p* < 0.01, ^∗∗∗^*p* < 0.001 in comparison to scopolamine treated group.

To determine retrieval of memory, all groups analyzed for effect on TRC on 22nd day, showed significant differences among all treatment groups [*F*(6,56) = 8.643, *p* < 0.0001] on the reference memory. As shown in **Figure 10**, significant decrease in TRC was observed in groups treated by donepezil (*p* < 0.001) and BuPC (400 and 600 mg/kg; *p* < 0.05 and *p* < 0.01, respectively) as compared to scopolamine treated group. Conversely, no significant change (*p* > 0.05) in TRC of ZnOPC (15 and 30 mg/kg) treated group was detected when compared to SCO group. Scopolamine injected group showed significant increase in TRC (*p* > 0.001) as compared to control group.

### Effect on Brain AChE Activity

**FIGURE 11:**
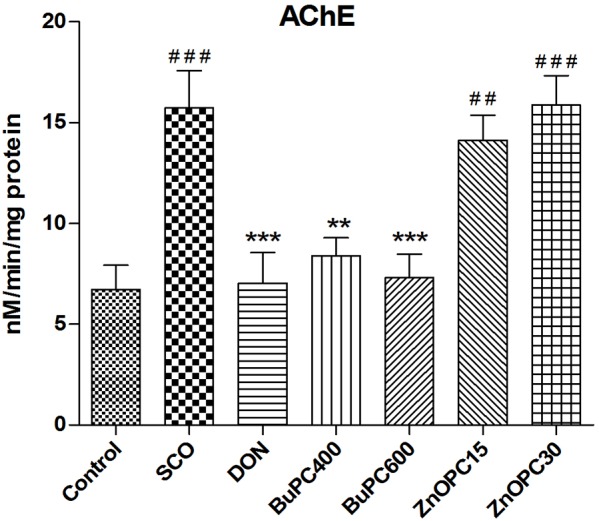
Effect of BuPC and ZnOPC on brain AChE activity. Data represented as mean ± SEM (*n* = 6), analyzed by one way ANOVA followed by Newman–Keuls multiple comparison test. Significant difference ^#^*p* < 0.05, ^##^*p* < 0.01, ^###^*p* < 0.001 in comparison to control group. Significant difference ^∗^*p* < 0.05, ^∗∗^*p* < 0.01, ^∗∗∗^*p* < 0.001 in comparison to scopolamine treated group.

Since BuPC antagonized the scopolamine-induced amnesia, its effect on AChE enzyme activity in brain homogenate was examined by using Ellman’s method. For the AChE specific activity estimated in the brain homogenates, significant overall differences between all groups [*F*(6,35) = 9.881, *p* < 0.0001] were observed (**Figure 11**). Significant (*p* < 0.001) increase in AChE activity in brain homogenate of scopolamine treated animals was observed in comparison to control group indicates marked destruction in memory. Scopolamine induced increase in AChE activity was significantly ameliorated by BuPC (400 and 600 mg/kg; *p* < 0.01 and *p* < 0.001, respectively) via restoration of cholinergic deficit. Results indicate inhibition of AChE activity with the administration of BuPC at both dose levels (400 and 600 mg/kg), whereas ZnOPC (15 and 30 mg/kg) was not able to significantly reverse the scopolamine-induced increased enzyme activity. Donepezil significantly (*p* < 0.001) reversed scopolamine induced increase in AChE activity.

### Biochemical Estimation

#### Effect on SOD, CAT, and GPx Activity

**FIGURE 12:**
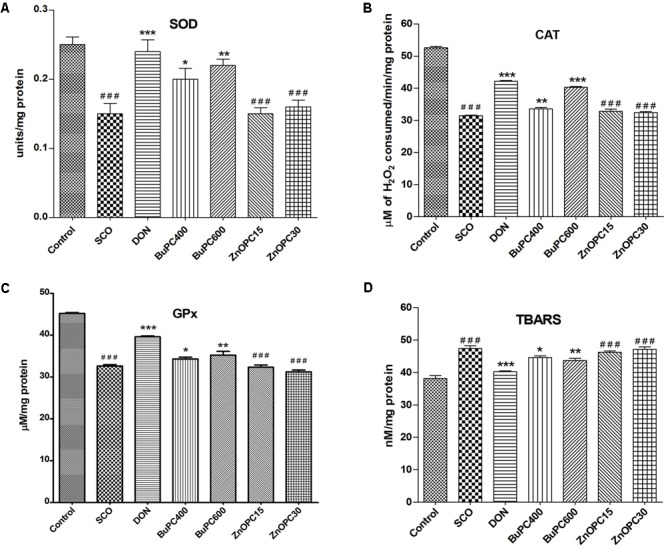
Effect of BuPC and ZnOPC on brain **(A)** SOD level, **(B)** CAT level, **(C)** GPx level and **(D)** TBARS level. Data represented as mean ± SEM (*n* = 6), analyzed by one way ANOVA followed by Newman–Keuls multiple comparison test. Significant difference ^#^*p* < 0.05, ^##^*p* < 0.01, ^###^*p* < 0.001 in comparison to control group. Significant difference ^∗^*p* < 0.05, ^∗∗^*p* < 0.01, ^∗∗∗^*p* < 0.001 in comparison to scopolamine treated group.

It has been reported that scopolamine directs an increase in oxidative stress by reducing the activity of antioxidant enzymes in the brain. For the SOD, CAT, and GPx specific activity estimated in the brain homogenates, significant overall differences between groups were found to be *F*(6,35) = 11.10, *p* < 0.0001; *F*(6,35) = 370.7, *p* < 0.0001; *F*(6,35) = 103.9, *p* < 0.0001, respectively (**Figures 12A–C**). The level of SOD, CAT and GPx activity was significantly (*p* < 0.001) decreased in SCO group as compared to control group. However, BuPC (400 and 600 mg/kg; *p* < 0.05 and *p* < 0.01, respectively) administered groups showed dose dependent improvement in scopolamine induced reduction in SOD and GPx activity in the brain homogenate. Whereas, BuPC (400 and 600 mg/kg, *p* < 0.01 and *p* < 0.001, respectively) administered groups showed dose dependent improvement in scopolamine induced reduction in CAT activity in the brain homogenate. Pre treatment with donepezil (*p* < 0.001) was most effective in restoring the enzyme activity decreased by the scopolamine. ZnOPC (*p* > 0.05), at both dose levels, could not reverse the scopolamine induced alteration in SOD, CAT, and GPx activity.

#### Effect on Lipid Peroxidation

For the lipid peroxidation level analyzed in the brain homogenates, significant overall differences between groups [*F*(6,35) = 28.72, *p* < 0.0001] were noticed (**Figure 12D**). As compared to control group, scopolamine treated mice significantly elevated (*p* < 0.001) TBARS levels in brain homogenate. Donepezil (*p* < 0.001) and BuPC (400 and 600 mg/kg; *p* < 0.05 and *p* < 0.01, respectively) substantially inhibited the scopolamine-induced elevated lipid peroxidation compared to SCO group. ZnOPC (*p* > 0.05), at both dose levels, was not found to be significant in decreasing the TBARS level elevated by scopolamine.

### Determination of Zn Content

**FIGURE 13:**
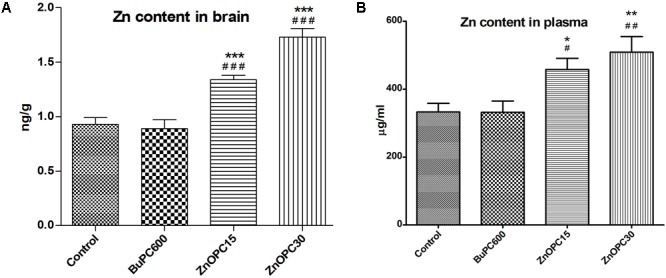
Effect of BuPC and ZnOPC on Zn level in **(A)** brain and **(B)** plasma. Data represented as mean ± SEM (*n* = 3), analyzed by one way ANOVA followed by Newman–Keuls multiple comparison test. Significant difference ^#^*p* < 0.05, ^##^*p* < 0.01, ^###^*p* < 0.001 in comparison to control group. Significant difference ^∗^*p* < 0.05, ^∗∗^*p* < 0.01, ^∗∗∗^*p* < 0.001 in comparison to scopolamine treated group.

Atomic absorption spectrometer was used to determine the total Zn concentration in brain and plasma of mice treated with BuPC, ZnOPC and normal saline. Results revealed concentration dependant significant increase in total Zn concentration in brain (**Figure 13A**) at 1.34 ± 0.08 ng/g (44%) and 1.73 ± 0.04 ng/g (86%) on exposure of ZnOPC 15 and 30 mg/kg, respectively, and similarly, plasma Zn concentration (**Figure 13B**) was observed to be 479 ± 33.12 μg/ml (37%) and 502 ± 45.70 μg/ml (50%). While no significant change was detected in BuPC600 group as compared to control group.

### Histological Alteration Studies

**FIGURE 14:**
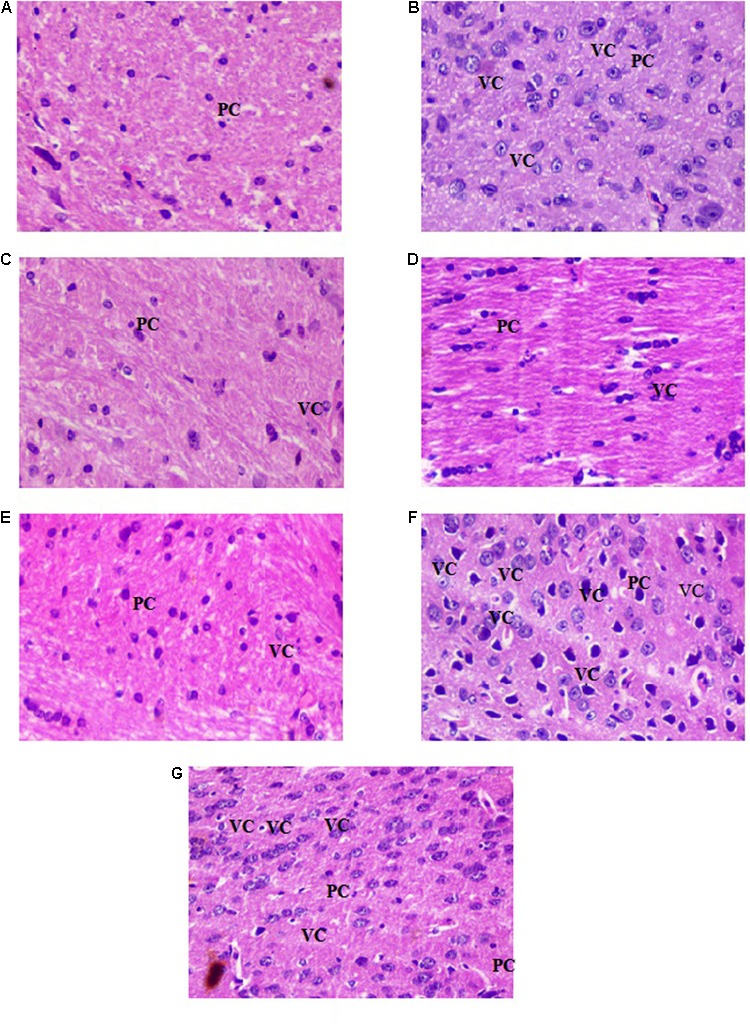
Photomicrographs of brain tissue stained with haematoxylin-eosin dye at 20× magnification **(A)** control group, **(B)** scopolamine group, **(C)** donepezil group, **(D)** BuPC400 group, **(E)** BuPC600 group, **(F)** ZnOPC15 group, and **(G)** ZnOPC30 group. PC-pyramidal cells; VC-vacuolated cytoplasms (index of neurodegeneration).

Histopathological examination of brain section illustrated the scopolamine mediated deleterious effects evidenced by elevation in vacuolated cytoplasm and focal gliosis, while pyramidal cells were diminished as compared to control group. Distinct reversal of scopolamine induced alterations in brain tissue was observed in BuPC pre administered groups. On the contrary, ZnOPC treated groups showed more number of dilated vacuolation and less number of pyramidal cells than scopolamine group, exhibits neurotoxic effect of ZnOPC (**Figures 14A–G**).

## Discussion

Current study was performed with the aim of evaluation of *in vitro* antioxidant and anticholinesterase inhibitory activity of different fractions of hydroethanolic extract of PC, a folklore nootropic plant. Since, according to cholinergic hypothesis of Peter Davies in 1976, there is a strong relationship between memory impairment and deficiency in brain choline among the people suffering from Alzheimer’s disease. This hypothesis suggests that disturbance of cholinergic system of the brain results in the gradual loss of cholinergic neurotransmission which ultimately leads to neuronal apoptosis, i.e., neuronal death (Ahmad et al., 2016; Singh et al., 2016). Imbalance in formation of reactive oxidative species and antioxidative defense system in the brain leads to affect the essential biomolecules such as enzymes, lipids, proteins, DNA and RNA which results in onset and further progression of age related neurodegenerative disease (Harman, 1993; Sadiq et al., 2015).

Our results revealed that the highest antioxidant and anticholinesterase potential showed by BuPC in concentration dependant manner. AChE inhibitory activity of BuPC was further examined kinetically by Lineweaver–Burk plot, which exhibited characteristic mixed kinetics attributed to various types of secondary plant metabolites. In addition, *K*_i_ was estimated to be 39.82 ± 1.17 μg/ml using Dixon plot, indicates competitive binding of BuPC fraction with ATCI. BuPC might bind at the site of substrate at AChE or conjugate at another site of AChE or conjugate with AChE-ATCI complex.

Plants, fruits, and vegetables rich in phenolic and flavonoid compounds exhibit antioxidant effect with nootropic and neuroprotective properties (Kumar and Khanum, 2012; Nile and Park, 2014). Results of total phenolic and flavonoidal content in current study also indicated their abundant existence in PC. Nootropic activity of methanolic extract of PC bark possesses antioxidant and AChE inhibition activity due to phenolic compounds (Bithu et al., 2012). Our study shows BuPC have the highest potential for antioxidant and AChE inhibitory activity evaluated by *in vitro* DPPH assay and Ellman’s method, respectively, as compared to other fractions, which might be associated with the presence of rich amount of phenolic and flavonoid compounds. Therefore, BuPC was further used for fabrication of ZnONPs.

Nanotechnology has become the most promising and rapidly growing technique over the recent years for the treatment of broad spectrum chronic diseases. Present study was envisaged for the first time to examine the comparative potential of BuPC and phytofabricated ZnOPC by continuous exposure for 21 days on scopolamine induced memory deficit mice.

Regarding oral toxicity study, Bithu et al. (2012) reported the safety of PC up to 2000 mg/kg, as there was no mortality and signs of toxicity in mice was observed by examining their behavioral status, such as convulsions, salivation, diarrhea, lacrimation and feeding behavior. No adverse effect was observed in vital organs, i.e., stomach, pancreas, eye and prostate gland, upto 31.5 mg/kg p.o. dose of ZnONP (Kim et al., 2014).

From our investigation, it has been discovered that PC, a traditional therapeutic plant, is able to reduce Zn acetate ions to ZnONP. Formation of ZnONP was outwardly confirmed from the color change. It might be because of the excitation of surface plasmon resonance in Zn oxide nanoparticles. UV absorption spectra of ZnOPC showed the absorption peak at 374 nm which signified the formation of ZnONP. DLS analysis of ZnOPC demonstrated slightly higher average particle size, 117.5 nm, as compared to FESEM analysis. FTIR spectra of ZnOPC confirmed the presence of phenolic, alcoholic, carboxylic acids, and amine groups. These functional groups proved the occurrence of various bioactive compounds, i.e., phenols, flavonoids, alkaloids, and terpenoids.

However, Zn content in brain and plasma was found significantly higher than control and BuPC treated animals, which reveals that ZnOPC seems to be absorbed in ionic form within the brain. Past investigation suggested ZnONP exposure rises Zn^2+^ mitochondrial and cytosolic concentration in cultured cells as well as rat white blood cells through dissolution of ZnONP in endosomes (Kao et al., 2012). Assimilated Zn^2+^ could partially lead the toxic effect of ZnONP in the vital organs of human beings and animals (Zhao et al., 2013).

Further, BuPC and ZnOPC were evaluated for their potential on spatial cognition in mice, subjected to memory deficit by i.p. injection of scopolamine, via behavioral models, antioxidant enzyme assay and AChE inhibitory activity. Scopolamine is a well known muscarinic cholinergic receptor antagonist, which leads to impairment in learning and memory process due to upregulation of acetylcholinesterase enzyme activity and oxidative damage in rodents (Bejar et al., 1999; Van Can et al., 2017). Cholinergic deficit and oxidative stress are reported as main culprit to aid into progression of age associated disorders like dementia (Zhao and Zhao, 2013; Adalier and Parker, 2016). On the basis of above reports, scopolamine induced amnesia is considered as a beneficial tool for the evaluation of potential of drugs as an antiamnesic agent. It has been previously reported that the herbal extract or any compounds reverse the amnesia induced by scopolamine have the potency to inhibit the activity of AChE enzyme (Jeong et al., 2008). Our results are in concordance with the previous recent reports that any substance which have AChE inhibitory and antioxidant activities could be considered as an ideal and potent agent for management of neurodegenerative diseases (Sadiq et al., 2015).

In behavioral models, EPM and HWM, mice administered with scopolamine exhibited significant increase in TL and TRC as compared to control group (**Figures 12, 13**). Memory impairment induced by scopolamine in mice was significantly attenuated by pretreatment with BuPC. But it was observed that ZnOPC treatment was not able to significantly reverse the scopolamine induced memory deficit. This is in well agreement with past study, which revealed chemically synthesized ZnONP damages the spatial learning and memory capability in rats through disruptive effect at the homeostasis of synaptic Zn concentration leading to direct hyperactive long-term potentiation and insufficient depotentiation in hippocampus (Han et al., 2011). In contrast to these results, it has also been reported that no steady difference was observed in emotional behavior of ZnOPC treated rats via EPM as compared to control group (Xie et al., 2012). The incongruity between current study and others is most likely due to variation in size, dose and treatment manner of ZnONP, as literature review suggested the toxic effect of chemically synthesized ZnONP after systemic distribution in different vital organs of animals varies with its mode of synthesis, surface chemistry, shape, size and stability (Cho et al., 2011, 2012; Li et al., 2012a,b; Vandebriel and De Jong, 2012). Past studies revealed *in vitro* and *in vivo* genotoxic, cytotoxic and neurotoxic effects of chemically synthesized ZnONP, but information in relation to *in vivo* toxicity of green synthesized ZnONP is still lacking (Shrivastava et al., 2014). Since we have used the phytofabricated ZnOPC in our study, it may be implicated that significant effect of plant extract on reversal of scopolamine induced memory impairment might be overcome by accumulation of Zn in brain as its turnover rate in brain is slow, which further leads to disturbance in homeostasis of brain Zn concentration.

To examine the responsible mechanism of action of BuPC and ZnOPC on SOD, CAT, GPx, TBARS level and AChE activity was evaluated *ex vivo* in brain homogenate of mice, as oxidative stress and AChE inhibitory activity are the main causative factor for the damage in spatial memory and learning. Several evidences revealed the possible connection of oxidative damage (increase in level of free radicals) with the neurodegenerative disease, i.e., Alzheimer’s disease (Bouayed et al., 2009). It has been demonstrated that SOD, CAT, and GPx specific activities, diminished in Alzheimer’s disease, are elevated with the use of antioxidants. Use of antioxidants leads to increase in concentration of SOD, which reduces the oxidative stress in tissue matrix by inhibiting the further formation of free radicals via dismutation of considerably toxic superoxide radicals into hydrogen peroxide and dioxygen radicals. It may be considered that resulted radicals of hydrogen peroxide catalyzed by increased CAT enzymes. An increase in GSH level, a thiol based endogenous antioxidant acts as a co substrate, accelerates the GPx assisted reaction by reduction of peroxides, such as lipid and hydrogen peroxides (Malik et al., 2013). It has been demonstrated that scopolamine associated increase in oxidative stress direct increased lipid peroxidation and reduced SOD, CAT, and GPx in rat brain, which causes memory impairment and neuronal cell death (Jeong et al., 2008; Pahaye et al., 2017). BuPC strongly antagonized the decreased SOD, CAT, and GPx induced by scopolamine in the mice brain. Index of lipid peroxidation and augmentation in TBARS activity has been considered as a key indicator of cognition damage in brain (Kwon et al., 2013). In addition, it has also been found that increased TBARS level in scopolamine administrated mice group was strongly reduced by BuPC. Our study is in consonance with the past studies showing significant increase in important enzymes (SOD, CAT, and GPx) level, and reduction in lipid peroxidation (TBARS) level, indicative of BuPC having significant antioxidant activity and possession of reducing the oxidative damage (Hritcu et al., 2015).

On the other hand, both doses of ZnOPC did neither significantly increase the diminished antioxidant enzymes level nor it lowered the increased TBARS level induced by scopolamine. Shrivastava et al. (2014) reported ZnONP induced oxidative stress in mouse brain leads to imbalance in neurotransmitter’s metabolism, which ultimately results in brain damage. Experimental evidence indicated that the accumulation of high concentration of Zn in brain passing through blood brain barrier responsible for neuronal injury, impairment of brain cognitive functions and further progression of neurodegenerative diseases (Campbell et al., 2005; Naziroglu et al., 2017). Disturbance in homeostasis of metal in brain and neuronal loss are both present in neurodegenerative diseases (Buzea et al., 2007). It has also been reported that after ingestion of ZnONP, Zn releases into its ionic form, i.e., Zn^2+^, which may penetrate into cells via ion channels due to its slight solubility, and later this, Zn^2+^ can lead to generate oxidative damage via production of great amount of reactive oxidative species and impairment in cell metabolism (Deng et al., 2009; Liu et al., 2013).

**FIGURE 15:**
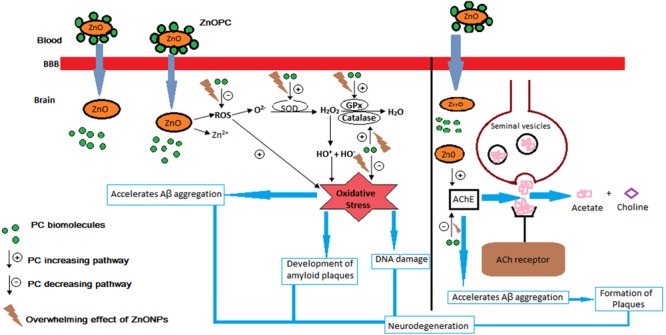
Proposed mechanism of neurodegeneration by ZnOPC.

Present findings showed BuPC significantly ameliorates upregulated AChE activity of brain homogenate in scopolamine treated mice. However, donepezil, a standard AChE inhibitor, showed the best results against scopolamine induced increase in AChE activity. It has been already reported that inhibition of AChE activity by plants and their constituents are effectively able to repair the memory impairment induced by scopolamine (Rahnama et al., 2015). BuPC might exhibit positive effect on deaggregation of β-amyloid tangles, since it alienate the effect of scopolamine and inhibit the AChE activity which are parallel to the report of Campos et al. (1998) and Liskowsky and Schliebs (2006), i.e., activation of β-amyloid precursor proteins followed by formation of tangles of β-amyloid (main component of formation of Alzheimer’s disease) due to scopolamine and AChE (Campos et al., 1998; Liskowsky and Schliebs, 2006). ZnOPC treated animal group did not significantly attenuate AChE activity increased by scopolamine. This may have bigger proposition since Zn has been proved to support *in vitro* aggregation of β-amyloid protein as well as development of amyloid plaques and neurotoxicity in the brain as the elimination rate of Zn from brain is very slow as well as concentration of extracellular Zn becomes high (Takeda, 2000). On the basis of results obtained in present study and previous reports the proposed mechanism for neurodegeneration by ZnOPC is illustrated in **Figure 15** (Mantyh et al., 1993; Bush et al., 1994; Han et al., 2011; Naziroglu et al., 2017).

## Conclusion

The current study depicts that preadministration with BuPC alleviate TL and TRC in scopolamine induced cognition deficit albino mice. It is potential *in vitro* antioxidant and AChE inhibitor, which found to be accordant with *in vivo* antioxidant assay and AChE inhibitory results. Our study recommends that BuPC exhibit strong neuroprotective activity by reducing *in vivo* oxidative stress and elevation in concentration of acetylcholine in mice brain. This fraction of PC might be a promising possibility for treatment of Alzheimer’s disease. Whilst, ZnOPC oral administration to albino mice for 21 days demonstrated accumulation of Zn^2+^ in the brain and plasma which devastate the neuroprotective effect of BuPC, polyphenolic rich fraction of PC. ZnOPC showed neurodegeneration by non-reversal of scopolamine induced oxidative stress, increased level of vital enzyme AChE and deprived performance of mice in behavioral models. Data of current experimental study is consistent with previous study that showed accumulation of Zn^2+^ ion in brain leads generation of reactive oxygen species (ROS) which results in cellular toxicity. Along with this, ZnONP exposure also grounds brain damage and neurotoxicity via disturbance in the normal metabolism of neurotransmitters, ultimately affecting long-term synaptic plasticity and causing imbalance between spatial cognition stability and flexibility. Even though green synthesized nature of ZnONP, it is potentially harmful and hazardous toward central nervous system. Hence critical evaluation is essential before wide utilization to guarantee safe use.

## Ethics Statement

This study was carried out in accordance with the recommendations of Committee for the Purpose of Control And Supervision of Experiments on Animals (CPCSEA) guidelines. The protocol was approved by the Institutional Animal Ethics Committee (IAEC), SHIATS.

## Author Contributions

EY performed the majority of experimental studies. DS and PY assisted in *in vitro* and *in vivo* studies. PY and EY analyzed and interpreted the data as well as drafted the manuscript. AV designed, supervised the study, and edited the manuscript. All authors contributed in the finalization of the manuscript.

## Conflict of Interest Statement

The authors declare that the research was conducted in the absence of any commercial or financial relationships that could be construed as a potential conflict of interest.
